# Evidence of exposure to henipaviruses in domestic pigs in Uganda

**DOI:** 10.1111/tbed.13105

**Published:** 2019-01-19

**Authors:** Christine Atherstone, Sandra Diederich, Hana M. Weingartl, Kerstin Fischer, Anne Balkema‐Buschmann, Delia Grace, Silvia Alonso, Navneet K. Dhand, Michael P. Ward, Siobhan M. Mor

**Affiliations:** ^1^ Sydney School of Veterinary Science The University of Sydney Camperdown New South Wales Australia; ^2^ International Livestock Research Institute Kampala Uganda; ^3^ Friedrich‐Loeffler‐Institut Institute of Novel and Emerging Infectious Diseases Greifswald ‐ Insel Riems Germany; ^4^ Canadian Food Inspection Agency National Centre for Foreign Animal Disease Winnipeg Manitoba Canada; ^5^ International Livestock Research Institute Nairobi Kenya; ^6^ International Livestock Research Institute Addis Ababa Ethiopia; ^7^ Institute of Infection and Global Health University of Liverpool Liverpool UK

**Keywords:** antibodies, Hendra virus, Henipavirus, Nipah virus, swine, Uganda

## Abstract

Hendra virus (HeV) and Nipah virus (NiV), belonging to the genus *Henipavirus*, are among the most pathogenic of viruses in humans. Old World fruit bats (family Pteropodidae) are the natural reservoir hosts. Molecular and serological studies found evidence of henipavirus infection in fruit bats from several African countries. However, little is known about the potential for spillover into domestic animals in East Africa, particularly pigs, which served as amplifying hosts during the first outbreak of NiV in Malaysia and Singapore. We collected sera from 661 pigs presented for slaughter in Uganda between December 2015 and October 2016. Using HeV G and NiV G indirect ELISAs, 14 pigs (2%) were seroreactive in at least one ELISA. Seroprevalence increased to 5.4% in October 2016, when pigs were 9.5 times more likely to be seroreactive than pigs sampled in December 2015 (*p* = 0.04). Eight of the 14 ELISA‐positive samples reacted with HeV N antigen in Western blot. None of the sera neutralized HeV or NiV in plaque reduction neutralization tests. Although we did not detect neutralizing antibodies, our results suggest that pigs in Uganda are exposed to henipaviruses or henipa‐like viruses. Pigs in this study were sourced from many farms throughout Uganda, suggesting multiple (albeit rare) introductions of henipaviruses into the pig population. We postulate that given the widespread distribution of Old World fruit bats in Africa, spillover of henipaviruses from fruit bats to pigs in Uganda could result in exposure of pigs at multiple locations. A higher risk of a spillover event at the end of the dry season might be explained by higher densities of bats and contact with pigs at this time of the year, exacerbated by nutritional stress in bat populations and their reproductive cycle. Future studies should prioritize determining the risk of spillover of henipaviruses from pigs to people, so that potential risks can be mitigated.

## 1 INTRODUCTION

Hendra virus (HeV) and Nipah virus (NiV) are highly pathogenic to humans (Chua et al., 2000; Murray et al., 1995). While both belong to the *Henipavirus* genus of the Paramyxoviridae family, the viruses have different host preferences and apparent geographic ranges. HeV first emerged in Australia in 1994 when 21 horses developed high fever and acute respiratory signs (Murray et al., 1995). Two people who had close contact with these horses were infected, one of whom died (Selvey et al., 1995). Since 1994, sporadic cases of equine HeV infection have been reported with seven human infections (four fatal) reported to date (World Health Organization, 2018). Fruit bats (*Pteropus* spp.) were determined to be the natural reservoir of HeV, with horses likely infected through contact with pasture or feed contaminated with fruit bat urine, feces, birthing products, or spat (fibrous plant material remaining after mastication by bats) (Mahalingam et al., 2012).

NiV was first discovered in 1998 following an outbreak of severe febrile encephalitis in humans in Malaysia (Chua et al., 2000). It was later found that a concurrent outbreak of NiV had occurred in pigs and that pig trading contributed to the spread of the outbreak between farms, including into Singapore (Mohd et al., 2000). This initial outbreak resulted in 265 cases of encephalitis in humans, and 105 fatalities, all of whom were involved in pig farming activities. To control the outbreak, more than 1 million pigs were culled. While pigs served as amplifying hosts of NiV in this Malaysia outbreak (Chua et al., 2000), Old World fruit bats (family Pteropodidae) were discovered to be the natural reservoir hosts of the virus (Chua et al., 2002). Subsequent outbreaks of NiV have occurred in India and Bangladesh and have been characterized by food‐borne and person‐to‐person transmission in the absence of apparent outbreaks in pigs (Chadha et al., 2006; Gurley et al., 2007; Islam et al., 2016; Luby et al., 2006). Nevertheless, the role of pigs in the original NiV outbreak led researchers to propose that pigs may be hosts to other henipaviruses, including HeV. To date, HeV has not been detected in domestic pigs on farms in Australia (Black et al., 2001) or elsewhere; however, they can be infected with the virus experimentally (Li, Embury‐Hyatt, & Weingartl, 2010).

Little is known about the ecology of henipaviruses in other parts of the world, including the potential for spillover into domestic animals. In Africa, henipavirus RNA has been detected in fruit bat feces in Ghana (Drexler, Corman, & Gloza‐Rausch, 2009) and fruit bat bushmeat in the Republic of Congo (Weiss et al., 2012). A full length African henipavirus sequence has also been described in fruit bats (Drexler et al., 2012). Further, serological studies on fruit bats in Cameroon (Pernet et al., 2014), Annobón Island in the gulf of Guinea (Peel et al., 2012), Ghana (Drexler et al., 2012; Hayman, Suu‐Ire, & Breed, 2008; Peel et al., 2013) as well as Tanzania and Uganda (Peel et al., 2013) have all found evidence of henipavirus exposure. There is some evidence that henipaviruses are circulating in pigs in Africa. In Ghana, about 5% of pigs sampled (*n* = 97) had detectable antibodies that showed cross‐reactivity with sHeV and sNiV G proteins in a Luminex‐based assay and in confirmatory Western blot. However these antibodies were non‐neutralizing in live virus neutralization assays (Hayman et al., 2011). In Nigeria, 20% of pigs were found seropositive for henipaviruses using indirect ELISA only (Olufemi, Umoh, Dzikwi, & Olufemi, 2015).

Pig farming is an increasingly important agricultural activity in parts of sub‐Saharan Africa, particularly in Uganda (Uganda Bureau of Statistics, 2008), driven by rapid growth in pork consumption (FAO Statistics Division, 2013) from increases in population, urbanization, and incomes (Delgado, 2003; Delgado, Rosegrant, & Meijer, 2001). The national pig population was estimated to be just 16,000 animals in 1961 (FAO Statistics Division F. and A. O. of the U. N., 2013), but has grown to 3.2 million in 2008, the year in which the most recent livestock census was completed (Uganda Bureau of Statistics, 2008). Millions of smallholder farmers rely on pig keeping to diversify their income, reduce financial risk, and improve livelihood security (Dione, Ouma, Opio, Kawuma, & Pezo, 2016). Traders link these smallholder farmers to consistent pork markets, aggregating live pigs from rural and urban farms and transporting them across multiple districts and regions within Uganda to meet the demand for pork in large urban areas (Atherstone et al., 2018). Despite the rapid growth in the domestic pig population, the extensive movement of pigs across the country and the widespread distribution of a suitable reservoir host for henipaviruses, the public health risk of spillover from pigs has not been investigated in Uganda. Therefore, the objective of this study was to determine if henipaviruses are present in pigs in Uganda and potential risk factors for this presence.

## 2 MATERIALS AND METHODS

### 2.1 Study area

The study area is described in detail elsewhere (Atherstone et al., 2018). Briefly, this study was conducted at Wambizzi Cooperative Society slaughterhouse in Nalukolongo, southwestern Kampala, which is the capital of Uganda. Wambizzi is the only formally registered pork slaughterhouse in Uganda. To meet the large urban demand for pork, live pigs are brought for processing from throughout the country. Because of this, the slaughterhouse is an ideal location for conducting disease surveillance, particularly for zoonoses, for which there is little information regarding the risk of pig farming and pork consumption on public health.

### 2.2 Study design

Findings reported here were part of a larger study conducted between December 2015 and October 2016 designed to demonstrate proof of freedom from filovirus infections. For this larger study, a target sample size of 157 in each of four discrete sampling periods was determined, corresponding with periods when there is a known increase in the number of pigs being processed to meet pork demand during national holiday seasons such as Easter, Independence Day and Christmas/New Years (Roesel, Holmes, & Grace, 2016). Thus, a total of 661 samples were available for analysis to assess exposure to henipaviruses. The sensitivity of the G ELISAs used in this study have not been determined given lack of availability of field sera (Fischer et al., 2018). Assuming a test sensitivity of between 80% and 95%, and an expected seroprevalence of not more than 5% (based on a previous study in Ghana) (Hayman et al., 2011), a sample size of between 115 and 97 was needed to demonstrate proof of freedom from henipaviruses with 99% confidence in each sampling period. Sample size calculations were performed using the online EpiTools application (Sergeant, 2018).

### 2.3 Selection of pigs and biodata collection

A systematic sampling strategy was used to select pigs for inclusion in this study. Since Wambizzi is not a mechanized slaughterhouse and has no slaughter line (Roesel et al., 2016), we physically counted pigs as they came through the door of the slaughter building and selected every 3rd pig for inclusion in the study. As pigs were selected, they were ear‐tagged with a unique identification number and this number was subsequently used to identify specimens collected from that animal. Biodata was collected using a standard form (Supporting Information Data S1). The form captured the date of sampling, ear tag number of the pig, rectal temperature taken at the time the animal was ear‐tagged, pig breed (based on visual classification of local, cross, or exotic), sex, whether the male pigs were intact or castrated, visible clinical signs of disease, and source location of the pig as reported by the pig trader. Sampling occurred over consecutive days, until the sample size for that sampling period was reached (*n* = 157).

A panel of samples was collected from each tagged pig. For purposes of this study, blood was collected in a clot activator vacutainer when the carotid arteries and jugular vein were cut. All samples were placed on ice in an ice box, stored for 2–3 hr until sampling was completed for the day and then transported to Makerere University College of Veterinary Medicine, Animal Resources and Biosecurity (COVAB), Kampala, Uganda, where they were placed under refrigeration until processing the following day. Serum was then separated by centrifugation (2,000x *g*), decanted into two cryovials (Sigma‐Aldrich, 2 ml), and stored at −80°C.

### 2.4 Detection of antibodies against Henipaviruses

All sera were initially tested in duplicates for antibody binding to sHeV glycoprotein (G) and sNiV G proteins using ELISA. Positive sera were retested in ELISA in duplicates to confirm initial results. Further, sera positive in either HeV G, NiV G, or both ELISAs, after initial and confirmatory rounds, was confirmed using Western blot against HeV nucleoprotein (N) antigen.

#### 2.4.1 Indirect ELISA based on sHeV and sNiV G proteins

sHeV and sNiV G proteins were produced and purified as described before (Fischer et al., 2016). Indirect ELISA based on sHeV G or sNiV G was performed as described elsewhere (Fischer et al., 2018). Briefly, proteins were diluted in 0.01 M PBS, pH 7.4, and coated onto Medisorp 96 well plates at a concentration of 100 ng/well (100 μl volume) at 4°C overnight. Extracts from untransfected *Leishmania tarentolae* served as mock antigens in control wells to evaluate unspecific binding of the sera. Plates were blocked with 5% skim milk in 0.01 M PBS for 2 hr at 37°C and washed three times with PBS/0.05% Tween‐20 (PBST). Sera were diluted 1:200 in 2.5% skim milk in PBST and added in duplicate to the control and antigen‐containing wells. After incubation at 37°C for 1 hr, the plate was washed and goat‐anti‐swine IgG HRP conjugate (Dianova) was added in a dilution of 1:10,000. After 1 hr incubation at 37°C, plates were washed and 3,3′,5,5′‐Tetramethylbenzidine (TMB) peroxidase substrate (Bio‐Rad, Munich) was added to the wells for colour development and stopped after 10 min at room temperature (RT) with equal amounts of 1 M sulphuric acid. Absorbance was measured at a wavelength of 450 nm against 590 nm in a Tecan Infinite 200Pro ELISA Reader (Tecan Deutschland GmbH). Samples with an optical density (OD) value of 0.35 or greater were considered positive.

#### 2.4.2 Western blot analysis of ELISA‐positive porcine serum samples

To confirm the specific binding of the ELISA‐positive porcine serum samples to the antigens, serum samples that tested positive during the initial and confirmatory screening on at least one ELISA were analysed for their reactivity in immunoblot, using baculovirus produced, and purified soluble HeV N antigen (kindly provided by Günther Keil, FLI). Briefly, N antigen was separated by 10% SDS‐PAGE and transferred to a nitrocellulose membrane. After transfer, the membrane was blocked in 5% skim milk in TBST overnight at 4°C. Then, the membrane was incubated with porcine sera (dilution 1:20 in 2.5% skim milk in 0.1% PBST) for 1 hr at 4°C. After several washes, species‐specific goat anti‐swine antibodies conjugated with HRP (Dianova) were incubated on the membrane for 1 hr at RT in a 1:5,000 dilution. The blot was then incubated with SuperSignal West Pico Chemiluminescent Substrate according to the manufacturer guidelines (Thermo Scientific), and protein detection was visualized. C70 serum (from an experimentally infected animal) in a dilution of 1:20 served as a positive control for the detection of HeV N antigen.

### 2.5 Plaque reduction neutralization test

To test if ELISA‐positive serum samples contained neutralizing antibodies, plaque reduction neutralization tests were performed as described previously using HeV and NiV (Weingartl et al., 2006). All procedures with live virus were performed under Biosafety Level 4 conditions at the National Centre for Foreign Animal Disease in Winnipeg, Canada.

### 2.6 Data analysis

After checking for typographical errors, data were exported into SPSS 24.0 (IBM Corp., Armonk, NY) for analysis. Standard descriptive analyses were conducted for categorical and continuous variables. For purposes of analysis, if pig sera had an OD value above the positive cutoff in both runs of that specific ELISA assay, they were considered seroreactive. Univariable logistic regression was performed to determine which variables were predictive of seroreactivity to henipaviruses. Explanatory variables with *p*‐value of <0.15 were fitted into a multivariable regression model to determine their association with henipavirus status.

For mapping, pig source locations (reported to the district level) were joined to the centroid of each district polygon in the 2014 Global Administrative Unit Layers for Uganda (Food and Agriculture Organization, Rome, Italy) using ArcGIS 10.2 (Environmental Systems Research Institute, Redlands, CA). The number of seroreactive pigs in each district was mapped using different symbols to denote sampling period.

### 2.7 Ethical considerations

Human and animal ethics approval for this research was obtained from the International Livestock Research Institute, Nairobi, Kenya (ILRI‐IREC2015‐01), the Ugandan National Council for Science & Technology (A499) and Makerere University College of Veterinary Medicine, Animal Resources and Biosecurity, Kampala, Uganda (SBLS.CA.2016). The Animal Ethics Committee at The University of Sydney, Australia, was also notified of external ethics approval (2015/891).

## 3 RESULTS

**Figure 1:**
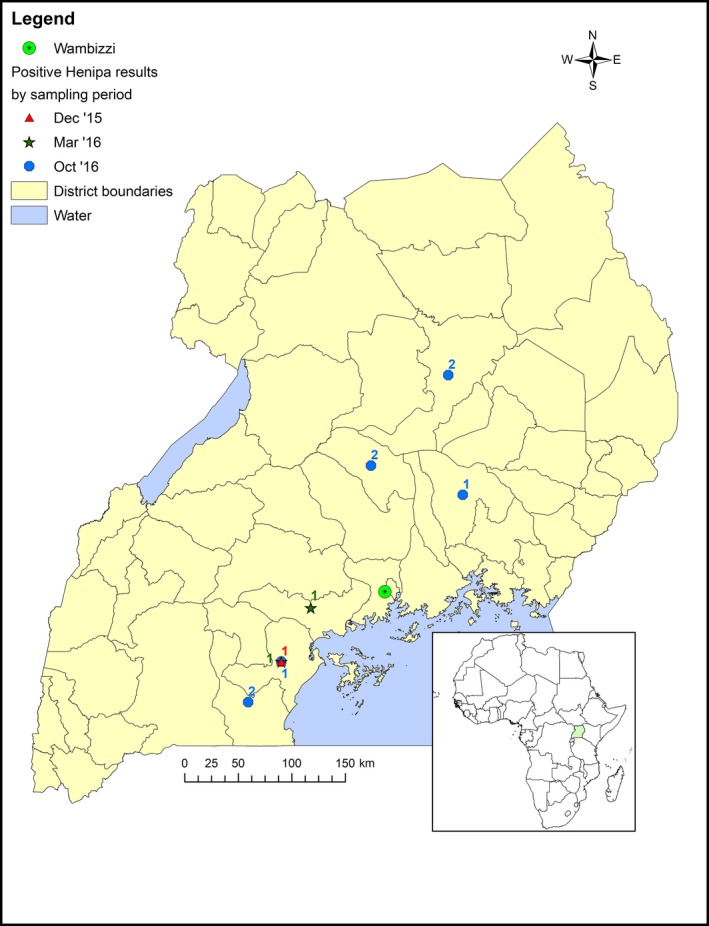
Source locations of henipavirus seroreactive pigs (*n* = 11) sampled at Wambizzi Cooperative Society slaughterhouse, Kampala, Uganda, 2015‐2016. The source locations of three seroreactive pigs was not recorded. [Color figure can be viewed at wileyonlinelibrary.com]

A total of 661 pigs were sampled, of which 14 pigs (2.1%) were seroreactive in at least one ELISA assay, after initial and confirmatory testing. The characteristics of sampled pigs are shown in Table 1 while source locations of seroreactive pigs are shown in Figure 1. While higher frequencies of seroreactivity were seen in female (2.3%; *p* = 0.44) and crossbreed pigs (2.8%; *p* = 0.29), as well as those sourced from the Northern region (14.3%; *p* = 0.02) or sampled in October 2016 (5.4%; *p* = 0.05), female and crossbreed pigs were not significant predictors of seroreactivity in univariable analysis. No seroreactive pigs had a fever at the time of sampling or visible clinical signs of disease. In multivariable analysis, the only significant predictor for henipavirus seroreactivity was pigs sampled in October 2016 (adjusted odds ratio: 9.5, 95% confidence interval: 1.1–80.0, *p* = 0.04; Table 1).

**Table 1:** Risk factors for Henipavirus seroreactivity from 661 pigs at Wambizzi Cooperative Society slaughterhouse, Kampala, Uganda, 2015–2016

Explanatory variables	Number	Number of seroreactive animals (%)	Outcome variable: Henipavirus seroreactive on at least one ELISA assay			
			Unadjusted odds ratio (95% CI)	*p*‐value	Adjusted odds ratio (95% CI)	*p*‐value
Sex						
Female	385	9 (2.3)	1.59 (0.49, 5.22)	0.44		
Male	270	4 (1.5)	1.00			
Not recorded	6	1	NA			
Breed				0.49^a^		
Cross	253	7 (2.8)	2.36 (0.49, 11.51)	0.29		
Exotic	231	4 (1.7)	1.46 (0.27, 8.08)	0.66		
Local	168	2 (1.2)	1.00			
Not recorded	9	1	NA			
Region				0.01^a^		
Central	360	8 (2.2)	1.00		1.00	
Eastern	112	1 (0.9)	0.39 (0.05, 3.20)	0.40	0.35 (0.04, 2.93)	0.34
Northern	14	2 (14.3)	7.33 (1.40, 38.29)	0.02	3.94 (0.69, 22.44)	0.12
Western	24	0	0.00	1.00	0.00	1.00
Not recorded	151	3	NA		NA	
Fever (>39.8°C)						
Yes	47	1 (2.1)	1.08 (0.14, 8.47)	0.94		
No	607	12 (2.0)	1.00			
Not recorded	6	1	NA			
Clinical signs						
Yes	16	0	0.00	1.00		
No	641	13 (2.0)	1.00			
Not recorded	4	1	NA			
Sampling period				0.01^a^		
December 2015	168	2 (1.2)	1.00		1.00	
March 2016	162	3 (1.9)	1.57 (0.26, 9.50)	0.63	1.76 (0.16, 19.77)	0.65
June 2016	163	0	0.00	1.00	0.00	1.00
October 2016	168	9 (5.4)	4.70 (1.00, 22.08)	0.05	9.50 (1.13, 80.02)	0.04

*Notes*

Explanatory variables with *p *< 0.15 in the univariable analysis were included in the final multivariable logistic regression model.

a Overall *p‐*value for non‐binary variables.

**Figure 2:**
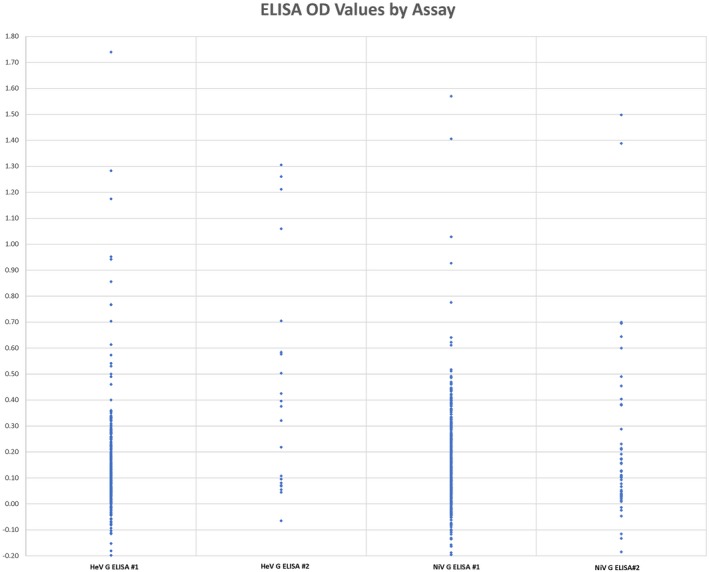
Optical densities of pig sera screened using HeV G and NiV G ELISAs [Colour figure can be viewed at http://wileyonlinelibrary.com]

**Figure 3:**
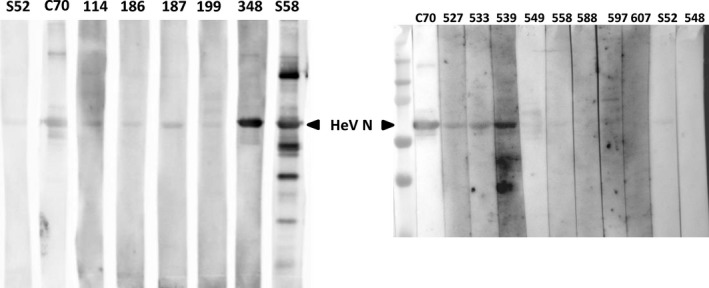
Western blot analysis of serum reactivity against HeV N. Porcine serum samples that exceeded the cut‐off in either sHeV G or sNiV G ELISA (*n* = 14) were tested for reactivity against purified HeV N antigen in a Western blot. C70 serves as positive control (serum from a NiV‐infected pig, S52 as a negative control [German pig]). All serum samples were diluted 1:20. [Color figure can be viewed at wileyonlinelibrary.com]

Optical density values for each ELISA assay are shown in Figure 2. While the positive cut‐off value was 0.35, several pigs (*n* = 5) had OD values >1.0 in duplicate runs of the ELISA assay. Eleven pigs (1.7%) were positive in both runs of the sHeV G ELISA while 11 pigs (1.7%) were positive in both runs of the sNiV G ELISA. Eight pigs (1.2%) were positive in both the sHeV G ELISA and sNiV G ELISA. Eight (1.2%) of the 14 serum samples that were positive in duplicate runs of at least one ELISA reacted with HeV N antigen in Western blot. Samples 186 and 199 showed only weak binding to the HeV N antigen whereas samples 114, 187, 348, 527, 533, and 539 reacted strongly with HeV N antigen in Western blot (Figure 3). However, none of the sera neutralized virus in the plaque reduction neutralization tests with either HeV or NiV.

## 4 DISCUSSION

This study is the first to report evidence of exposure of the Ugandan pig population to henipaviruses or henipa‐like viruses. Pigs in this study were sourced from many farms throughout Uganda, suggesting multiple, albeit rare, introductions of henipaviruses or henipa‐like viruses into the Ugandan pig population. We postulate that given the widespread distribution of the African fruit bat *Eidolon helvum* in Uganda (Mickleburgh, Hutson, Bergmans, Fahr, & Racey, 2008), spillover of henipaviruses from fruit bats to pigs could result in exposure of pigs at multiple locations. However, it should be noted that the previous report of henipavirus infection in *E. helvum* in Uganda was based on a very small sample size (one pooled sample) (Peel et al., 2013). Uganda does fall within the geographic range of fruits bats of the Pteropodidae family (World Health Organization, 2008), which to date are the only known reservoirs of henipaviruses (Daszak et al., 2011).

Pigs sampled in October 2016 were 9.5 times more likely to be seroreactive than pigs sampled in December 2015 (*p* = 0.04). Uganda experiences bi‐modal rainfall with intermittent rains between October and December. Clustering of fruit bats around food resources, may lead to pigs being exposed to contaminated bat urine and faeces or by eating dropped fruit. Virus contaminated bat saliva deposited on fruit has been shown to be the source of infection during the NiV outbreak in Malaysia (Chua et al., 2000; Yob et al., 2001) and the HeV outbreaks in Australia (Field et al., 2001). This route of transmission has also been speculated as a possibility for several other emerging viruses (Plowright et al., 2016; Pourrut et al., 2009). However, the reason pigs sampled in October were more likely to be seroreactive might be due to other unrecognized henipavirus emergence factors. Research has identified higher densities of specific bat species (Martin et al., 2016; Páez et al., 2017), increased contact around scarce food resources during drier weather (Páez et al., 2017), periods of nutritional stress (Plowright et al., 2008), and increased numbers of reproducing females (lactating and near‐term pregnant) (Plowright et al., 2008) as contributing to higher risk of infection in bat hosts and thus virus spillover into domestic animals.

When compared to the Central region no other regions were significantly associated with seroreactive status in pigs. Central region was used as the reference category in the regression analysis because this region has the highest pig population in Uganda (Uganda Bureau of Statistics, 2008). However, Northern region did have a much higher frequency (14.3%, 95% CI: 1.40, 38.29) of seroreactive pigs than other regions. The small number of pigs sampled from this region likely had an impact on the statistical power to determine if pigs in the Northern region are more at risk of henipavirus exposure. In the future, more pigs from the Northern region should be sampled to determine if pigs from this region are more likely to be exposed to henipavirus(es).

The failure to identify VNT positive sera in this study may be due to several reasons. It is possible the virus(es) circulating in Uganda are sufficiently divergent such that they do not neutralize the HeV or NiV used in the live virus assays. Multiple henipavirus‐related sequences have been reported in *E. helvum* (Baker et al., 2012; Drexler et al., 2009, 2012; Peel et al., 2013), indicating a considerable diversity of henipaviruses in Africa. In addition, because nothing is known about the duration or stability of antibody response in pigs, it is possible that neutralizing antibodies are less stable or detectable for a shorter period of time post‐infection than antibodies against other antigens. Failure to detect neutralizing antibodies is in line with similar serological studies in pigs in Ghana (Hayman et al., 2011) and Bangladesh (Chowdhury et al., 2014). Similarly, neutralizing antibodies were only detected in some of the seroprevalence studies in fruit bats in Africa, namely Ghana (Hayman et al., 2008; Peel et al., 2013) and Tanzania (Peel et al., 2013).

Finally, this serological study in apparently healthy animals provides little insight into the clinical consequences of henipavirus infections in pigs. None of the seroreactive pigs had visible clinical signs at the time of sampling. This may suggest that the henipaviruses circulating in Uganda are apathogenic, similar to findings from animal infection studies involving Cedar virus (Marsh et al., 2012). Alternatively, affected animals may have had signs of disease earlier, which had abated by the time of sampling.

Overall, this study extends the findings from Ghana (Hayman et al., 2011) and Nigeria (Olufemi et al., 2015) and confirms that pigs in Uganda are exposed to henipaviruses. Future studies should prioritize determining the risk of spillover of henipaviruses from pigs to people so that any potential risks can be mitigated.

## CONFLICT OF INTEREST

All authors declare that they have no competing interests.
