# Supplementary material for: Evidence of exposure to henipaviruses in domestic pigs in Uganda
Source: Transbound Emerg Dis. 2019 Jan 19;66(2):921–8. doi: 10.1111/tbed.13105 (PMC6849855; doi:10.1111/tbed.13105)
Supplement: Supplementary file 1 [file TBED-66-921-s001.pdf]

ILRI
INTERNATIONAL
LIVESTOCK RESEARCH
INSTITUTE

THE UNIVERSITY OF SYDNEY

Wambizzi Sampling: Pig Biodata

1. Date (DD/MM/YY): ___/___/___

2. Ear tag ID (identical to sample ID whole blood, serum, fecal sample, nasal swab, urine sample): ____________

3. Rectal temperature °C __._

4. Pig breed: local ☐
   exotic ☐
   cross ☐

5. Gender: male ☐ male intact Y / N
   female ☐

6. Visible symptoms of disease (circle all that apply):

1=diarrhoea	2= anorexia	3=dullness	4=swaying gait	5=skin flash	6=respiratory problems
7=vomiting	8=coughing	9= shivering	10=foaming at mouth	11=wounds	12=Other

If other, please explain:

____

____

7. Pig source location (village, subcounty, district):

____

8. Gross pathology noted:

____

____

____

____

Barcode for:

a. epididymis sample___________________________

b. liver sample_________________________________

c. lung sample_________________________________

d. placenta sample_____________________________

e. spleen sample_______________________________

f. Tracheobronchial lymph nodes_________________
